# A compact targeted drug delivery mechanism for a next generation wireless capsule endoscope

**DOI:** 10.1007/s12213-016-0088-9

**Published:** 2016-05-13

**Authors:** Stephen P. Woods, Timothy G. Constandinou

**Affiliations:** Imperial College London, South Kensington, London, UK

**Keywords:** Biomedical robotics, Gastrointestinal (GI) tract, Capsule endoscopy, Targeted therapy, Microrobot, Wireless capsule endoscopy

## Abstract

This paper reports a novel medication release and delivery mechanism as part of a next generation wireless capsule endoscope (WCE) for targeted drug delivery. This subsystem occupies a volume of only 17.9mm^3^ for the purpose of delivering a 1 ml payload to a target site of interest in the small intestinal tract. An in-depth analysis of the method employed to release and deliver the medication is described and a series of experiments is presented which validates the drug delivery system. The results show that a variable pitch conical compression spring manufactured from stainless steel can deliver 0.59 N when it is fully compressed and that this would be sufficient force to deliver the onboard medication.

## Introduction

Endoscopes and colonoscopies are used routinely by gastroenterologists to diagnose and treat pathologies such as Crohn’s disease in the gastrointestinal (GI) tract. However the small intestine poses a problem for these conventional methods as it is very difficult to access. One method employed to overcome this problem is the use of wireless capsule endoscopes (WCE). These pill-sized cameras take pictures of the intestinal wall which are then used to diagnose pathologies. The market leader in WCE is Given Imaging Ltd. [1] who in 2000 specifically designed the M2 A to examine the small intestinal tract through natural peristaltic pressure. The 11.0 mm in diameter X 25.0 mm long pill contains a CMOS-based image sensor to take pictures of the intestinal wall. The pictures can be transmitted to a device worn by the patient for later evaluation. A detailed review of WCE is reported in [2].

The problem with current WCE devices is that they do not offer the ability to administer therapy to an affected area of the GI tract. However there is a clinical need to target and treat these pathologies [3] hence endeavours have been made by researchers to perform regional drug absorption services. These systems are capable of delivering up to 1 ml of medication to a region of the GI tract such as the jejunum, ileum, ascending colon or descending colon either through the progressive release of medication over a period of time or through releasing in a bolus form. However the delivery methods employed by these devices prevent the direct targeting of specific pathogens such as tumours or ulcers as the medication is spread over a section of lumen due to the constant movement from peristalsis, further they have no means of stopping and holding their position.

### State of the art

The focus of conventional passive WCE medical devices has been to take pictures of the GI tract however the next generation of WCE must possess additional functionality to enable them to carry out useful tasks such as performing a biopsy or targeted drug delivery [4]. To facilitate this additional functionality active mechanical parts will be required to perform the tasks such as resisting natural peristalsis.

#### Resisting peristalsis

The limitations of conventional WCE have led researchers to explore methods of increasing a capsule’s functionality such as the ability to navigate through the GI tract [3–5] or resist natural peristalsis [6–10]. The paddling based microrobot developed by Park et al. (2007) [11] was developed specifically for navigating the GI tract. This method of locomotion uses a leadscrew engaged with six radially positioned legs to propel a microrobot forwards. A three-legged anchoring mechanism which utilises micropatterned adhesives for resisting peristalsis is proposed by Glass et al. (2008) [12]. This method of anchoring to the intestinal wall reduces the risk of injury to the intestinal tissue.

Menciassi et al. (2005) [13] propose a clamping system utilising three grasping units which extend forwards from the capsule and are driven by thermally activating a 100 μm diameter SMA for the purpose of clamping to the wall of the GI tract to monitor pH levels long-term.

A major limitation of commercial WCEs is the lack of control over their speed and direction of travel as they rely on the natural movement from peristalsis to progress through the GI tract. A potential solution to this issue is the robotic legged locomotion device developed by Valdastri et al. (2009) [14]. The 12-legged endoscopic capsular microrobot utilises two sets of six legs which are integrated into the capsule. The legs allow the capsule to navigate through the GI tract and allow for better visualisation due to the legs uniformly distending the collapsed colon tissue.

Inspecting the lining of the oesophagus is routinely performed using a flexible endoscope however this procedure results in discomfort for the patient. An approach proposed by Tognarellia et al. (2009) [15] utilises three equally spaced SMA flaps incorporated into an 11 mm in diameter by 31 mm long capsule to rapidly halt the progress of an oesophageal WCE for the purpose of inspecting the oesophagus lining.

#### Drug delivery

Phaeton Research has developed the Enterion capsule [16] for the purpose of delivering drugs to a specific location in the GI tract. The 11 mm in diameter by 32 mm long capsule has an onboard drug reservoir capable of holding 1 ml of medication in either a liquid, powder or solid form. The capsule relies on natural peristalsis to progress through the alimentary canal. Tracking the capsule’s position through the GI tract is by means of a gamma-emitting radionuclide placed inside the capsule and a gamma camera. Once a target site has been located the medication is ejected through the release of a compressed spring which operates a piston that pushes the medication out of a port at the rear of the capsule.

The InteliSite capsule manufactured by Innovative Devices LLC [17] delivers 1 ml of medication in either a liquid or a powder drug form through side ports which are activated when the capsule is exposed to a radio frequency magnetic field. The magnetic field causes two SMA wires which are connected to a sleeve to heat up and straighten out resulting in the sleeve rotating and aligning the side ports to release the medication. The 10 mm diameter by 32 mm long capsule disperses the medication by virtue of the turbulence created by the capsule travelling through the GI tract.

The IntelliCap is an intelligent pill developed by Philips Research [18] to electronically control the release of drugs into the GI tract. The 11 mm diameter by 26 mm long pill integrates a drug reservoir, battery, temperature sensor, pH sensor, RF wireless transceiver, fluid pump and microprocessor. The microprocessor controls the release of medication through the fluid pump which allows the medication to be released in either a burst, progressive release or a multi-location dosing form.

Karargyris et al. (2015) [19] propose the OdoCapsule which provides video stability and positional information while navigating the GI tract. The system, which is based on previous work carried out by Bourbakis et al. (2010) [20], utilises three positional sensor wheels as opposed to the one wheel approach proposed by Lambert et al. (1991) [21]. The capsule’s three sensor wheels are mounted on 15 mm long legs which protrude out of the 13 mm diameter by 30 mm long capsule by 35° due to torsion springs. The wheels contact the GI tract wall and each rotation of the wheel is recorded to monitor the progress of the capsule.

#### Localization

It is of great benefit to know the position and orientation of the WCE when it is travelling through the GI tract [22] as the 3D positional data can be used to return to a site of interest to perform further diagnosis or to administer therapy. The mechanical method of employing wheels proposed by Lambert et al. [21] and Karargyris et al. [19] may offer a potential solution however relocating the site of a previously captured image, in relation to the position and orientation of the capsule, is still a challenge to achieve. Further the unrestrained, slippery, compliant environment of the small intestine adds greater complexity to the problem. Therefore alternative methods of tracking a capsule’s position which do not use a mechanical means of interaction with the GI tract wall have been proposed by researchers.

The InteliSite capsule [17] can be tracked through the GI tract using gamma scintigraphy which will detect Indium (111 In) or Technetium (99 mTc) gamma isotopes which have been incorporated into the capsule. This method of localization is limited as only the capsule’s general position can be determined.

The position of the M2 A by Given Imaging [1] is determined by monitoring electromagnetic waves transmitted to eight external receivers [23]. The M2 A utilises radio frequency triangulation to monitor the signal strength of the transmitted images to monitor the progress of the capsule through the GI tract. This method is restricted to 2D positioning and a positional error of 37.7 mm [24].

A promising method to monitor the movements of a WCE through the GI tract is through the magnetic field strength of an onboard permanent magnet [23]. Magnetoresistive sensors attached to the skin allow the orientation and position of the WCE to be approximated with average positional errors of 3.3 mm reported [25].

### Research aim

The aim of this research is to increase the capability of passive capsules by increasing their functionality, specifically to achieve targeted drug delivery in the GI tract. Our previous work focused on developing a mechanism to resist natural peristalsis and also to target specific locations in the GI tract. A novel biologically-inspired holding mechanism was proposed which is based on the order of insects known as Coleopterans (beetles). Coleopterans have two pairs of wings and include insects such as the coccinellidae (ladybird). The ladybird has the ability to fold away its wings behind a second hardened pair of wings which are used as a casing to protect them. Using the concept of a pair of folded wings protected by an outer case as a basic premise, a compact holding mechanism was prototyped from Nylon 6 and incorporated into a WCE for the purpose of resisting natural peristalsis in the GI tract [26]. To further increase the functionality of WCE a novel targeting mechanism was proposed which can be incorporated into a WCE for the purpose of delivering a metered dose of medication to a targeted site of interest in the small intestinal tract. A fully functioning prototype targeting mechanism was presented which is capable of positioning a needle within a 360 degree envelope [27].

This paper presents the final mechanism required for targeted drug delivery in the GI tract. A method of delivering medication from the microrobot’s onboard reservoir to a site of interest in the small intestinal tract is presented which, combined with the holding mechanism and targeting mechanism, results in a microrobot with sufficient functionality to deliver a metered dose of medication to a target site in the GI tract. The paper is organised as follows: Section 2 introduces the concept design, Section 3 describes the drug delivery system, Section 4 presents validation of the drug delivery system through experimentation and Section 5 concludes this work.

## Concept design

**Fig. 1:**
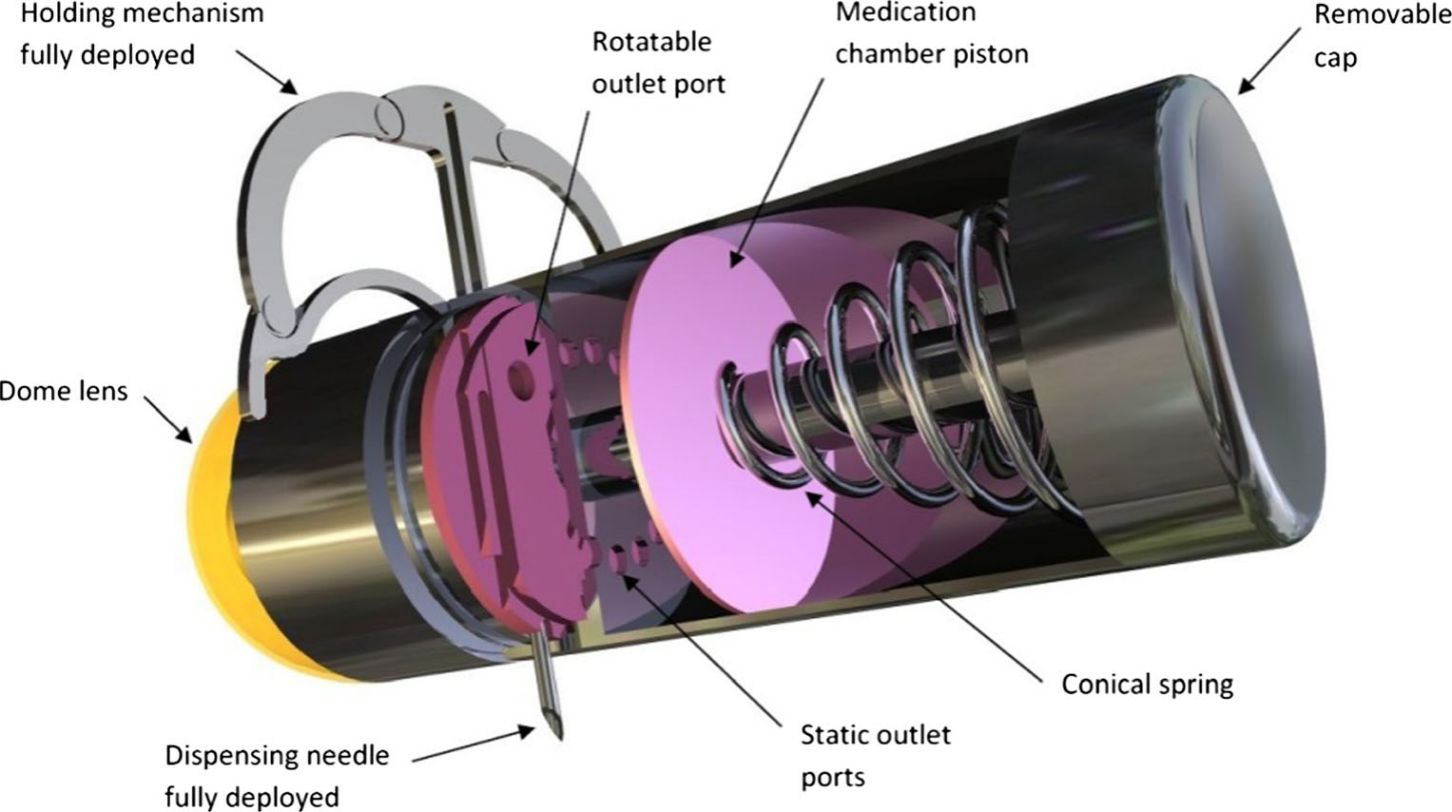
Microrobot concept design capable of delivering 1 ml of targeted medication. Needle shown fully extended and the medication partially deployed

The clinical need to treat pathologies of the GI tract are not being met by today’s technology. Currently pathologies such as Crohn’s disease and ulcerative colitis are being treated by conventional endoscopes in the lower section of the alimentary canal however the middle section of the GI tract, the ilium and jejunum, can only be accessed by the application of a WCE. Images transmitted from a WCE are reviewed to diagnosis pathologies however these passive devices are limited as they are not capable of administering curative treatments. Figure 1 shows a concept design of a microrobot with integrated systems to facilitate targeted drug delivery in the GI tract.

Figure 1 shows a microrobot concept design capable of resisting natural peristalsis through the deployment of a holding mechanism, targeting a pathology of the GI tract and delivering a 1 ml dose of medication to the site of interest through the positioning of a needle. The needle has the ability to be positioned in a 360 degree envelope while simultaneously maintaining a diametrically opposite relationship with the holding mechanism, this novel feature guarantees needle penetration of the GI tract wall.

**Fig. 2:**
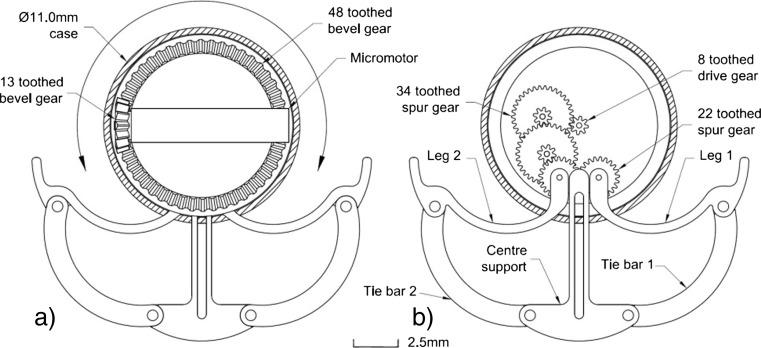
Holding mechanism design showing: 0.2 module 13 tooth and 48 tooth bevel gears set a) and central 0.1 module 8 tooth spur gear is driven by the 48 teeth bevel gear

The holding mechanism in Fig. 2 uses a single micromotor manufactured by Faulhaber to open and close two legs. The two legs are connected to a central support via two pinned leg ties. The two-legged design utilizes a micromotor, which is orientated in a vertical position. The micromotor is connected to a bevel gear set, which allows the micromotor’s rotation to be translated through 90 degrees. The bevel gear drives a gear train of spur gears which drive the legs in and out. This novel configuration reduces the micromotor’s revolution per minute (RPM). The reduction in RPM will result in a multiplication of the micromotor’s torque; this will give the legs the strength required to distend the GI tract wall and hold the microrobot in place. A detailed evaluation of the holding mechanism can be found in [26].

**Fig. 3:**
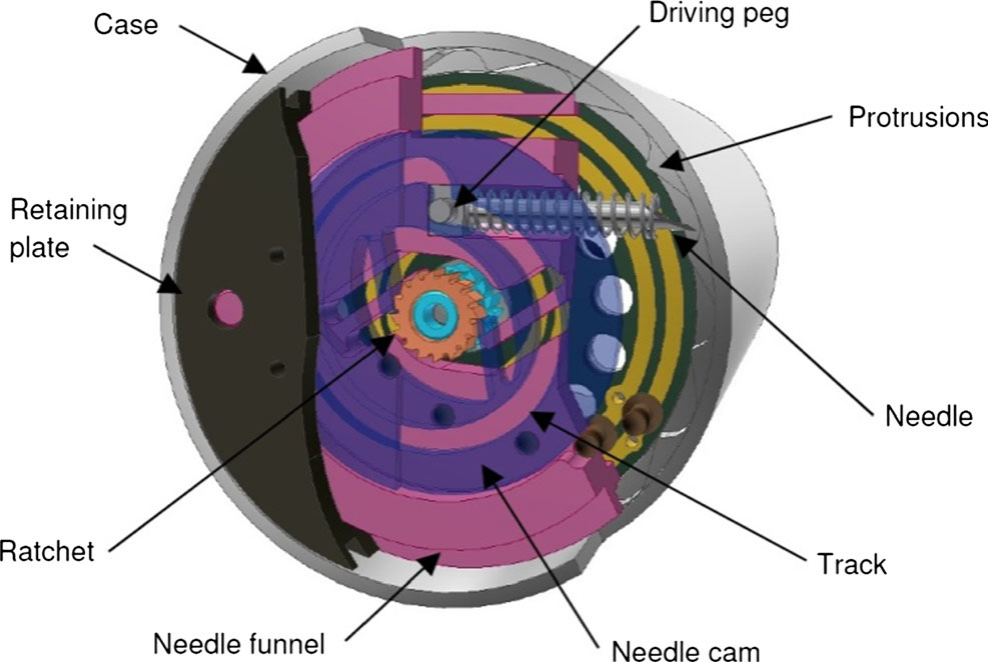
Section view through the component parts of the needle positioning mechanism. Material removed for clarity and needle shown fully extended

Delivering a metered dose of medication to a site of interest is achieved through a novel mechanism utilising a cam, a needle funnel, two opposing ratchets, a needle and a single micromotor manufactured by Namiki. Orientating the micromotor along the central axis of the microrobot allows for simple coupling to the cam and needle funnel via two opposing ratchets. Driving the micromotor anticlockwise allows for the angular positioning of the needle, which has been fixed at one of 16 positions equally spaced. Driving the micromotor clockwise extends and retracts the needle. The novel positioning mechanism functions through the use of a series of ratchets engaging and disengaging to allow the various operations to be performed. A detailed evaluation of the targeting mechanism can be found in [27]. Figure 3 shows a section view through the component parts of the needle positioning mechanism.

## Drug delivery system

The drug delivery system has the capacity to store a 1 ml volume of medication onboard the microrobot. The force generated by a compressed conical spring is proposed to expel the medication. The conical spring engages with a piston and when activated it will force the medication out of one of sixteen preset holes at the base of the medication chamber. The holes have a thin membrane across them to protect the integrity of the medication and to prevent the medication from leaking into the needle positioning mechanism. Rotating the needle positioning mechanism will align a port on the back of the needle funnel with one of the sixteen holes, the remaining holes would automatically be blanked off and therefore prevent any ingress of the medication. The novel configuration of the conical spring provides a compact design however a triggering mechanism will also be required to automatically release the spring.

The following sections discuss the design and analysis of the drug delivery system, the trigger mechanism and the issue of power distribution through the microrobot. A series of experiments are presented which validate the drug delivery system.

**Fig. 4:**
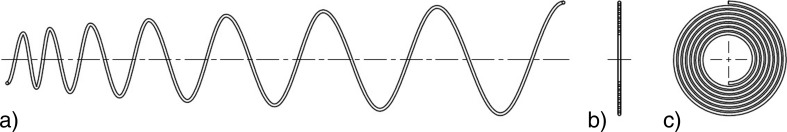
Conical compression spring configurations, 7.5 coils fully expanded with a variable pitch a) collapsed section view b) and fully coiled position c)

Using a compressed spring to drive the delivery of the medication would require allocating a volume of space within the microrobot for the spring’s solid state volume and for the spring’s actuation mechanism. A novel way of minimising the spring’s volume is to use a conical compression spring, Fig. 4.

Figure 4 shows a variable pitch conical compression spring in its free state a) in the primed position b) and a plan view in the fully coiled position c). Winding the coils such as they lie inside each other (Fig. 4c) enables the same number of coils to be used as in a conventional compression spring to generate a force, however it significantly reduces the space required to hold the spring. For example, a conical compression spring with six Ø0.5 mm coils would occupy a solid state volume of 40.1mm^3^ this can be compared to a volume of 55.0 mm^3^ for a conventional compression spring with the same geometry. Providing clearance between the coils enables them to be spiralled inside each other. A variable pitch is required between each coil to allow the spring to collapse at the same rate, this is due to the larger coils collapsing faster than the tighter coils. This configuration allows the active coils to bottom simultaneously resulting in a straight line force versus deflection curve similar to the output of a conventional compression spring. To release the spring from the compressed position a trigger mechanism will be required.

The conical compression spring will require analysing to determine the optimum medication delivery force. This will involve analysing a number of spring variables such as the number of coils, the diameter of the wire and the force required to operate the system. All these factors will influence each other’s performance and a balance will need to be determined which will minimise the space required to house the system.

### Medication delivery

To deliver a 1 ml volume of medication to a target site requires the onboard supply of medication to be pushed through a series of holes allowing it to be expelled through the needle tip. To achieve this task an evaluation of the force required to expel the medication must be performed. The bore of the needle is the most restrictive section of the delivery system for the flow of medication. The needle has an internal diameter of 0.38 mm and a length of 6.7 mm. As the needle has a consistent diameter and length Poiseuille’s equation can be used to model the flow of medication through the needle. The assumptions which will be made with Poiseuille’s equation are that the medication is incompressible and Newtonian, the flow is laminar and friction will be ignored [28].

The flow rate of a needle ${Q}_f$ can be calculated as follows:

1 $$ {Q}_f=\frac{\pi {r}^4\left({P}_2-{P}_1\right)}{8{n}_vl} $$

Where *r* is the radius of the needle’s bore, *n*_*v*_ is the viscosity of the medication and *l* is the length of the needle. ${P}_1$ is the pressure at the end of the needle and *P*_2_ is the pressure required to expel the medication. For the purpose of analysis the viscosity of the medication will be based on water at 36 °C (0.705 × 10^−3^ Pa s). ${P}_1$ is the pressure resistance of the medication being introduced into the GI tract wall. The interstitial hydrostatic pressure in subcutaneous tissue can range from -8 mmHg to +6 mmHg for normal tissue however for tissue with a tumour the pressure can be much greater. Therefore ${P}_1$ will be taken as the subcutaneous pressure in a tumour which is approximately +20 mmHg (2.6 kPa) [29].

As the medication must be absorbed by the subcutaneous tissue a delivery time has been set at 3.5 s. The flow rate (${Q}_f$) can be calculated by dividing the 1 ml volume of medication stored onboard the microrobot by the target delivery time (3.5 s).

Rearranging equation (1) allows the pressure difference $\varDelta P$ to be calculated (2.6 kPa). As the pressure difference ΔP = *P*_2_ − *P*_1_ the pressure required to deliver the medication in 3.5 s $\left({P}_2\right)$ can be determined, 5.2kPa. The delivery pressure $\left({P}_2\right)$ multiplied by the area of the medication chamber’s plunger gives the delivery force required to expel the medication. The force applied to the medication chamber’s plunger equates to 0.43 N.

The flow of the medication through the needle was assumed to be laminar however to confirm this the Reynolds number can be used to determine how the medication flow is acting:

2 $$ {R}_e=\frac{\rho dv}{n_v} $$

Where *ρ* is the density of the medication, *d* is the diameter of the needle, *v* is the velocity of the medication and ${n}_v$is the viscosity of the medication.

The Reynolds number for the system equates to approximately 1.9. For ${R}_e$ < 2100 the flow is laminar and for ${R}_e$ > 4000 the flow is turbulent. A Reynolds number between these two limits results in transition flows which can either be laminar or turbulent. The low Reynolds number confirms the flow will be laminar and therefore no compensation for changes in pressure will be required.

There are a number of variables which can affect the delivery time, for example if the viscosity of the medication were to increase it would slow the delivery speed while an increase in temperature would speed up the delivery time.

### Spring design analysis

The geometry of the microrobot places limits on the allowable design parameters which can be used in the design of a conical spring. However the spring must be designed to fit within a volume of 40.0 mm^3^ and deliver a force of 0.43 N over a stroke of 12.0 mm. As the spring’s deliverable force is known the spring’s deflection *f* can be calculated by the following formula for conical compression springs:

3 $$ f=\frac{16n\left({r}_1+{r}_0\right)\left({r}_1^2+{r}_0^2\right){F}_d}{d^4G} $$

Where *n* is the number of active coils, ${r}_0$ is the mean coil radius of the first coil and ${r}_1$ is the mean coil radius of the last coil, ${F}_d$ is the force, *d* is the wire diameter and *G* is the module of rigidity. A stainless steel wire spring with a module of rigidity value of 69.0GNm^−2^ [30] will be used for the spring analysis.

Using an input force of 0.43 N and a combination of 0.25 mm diameter wire, 7.5 active coils, a mean coil radius of 2.1 mm for the first coil and a mean coil radius of 5.0 mm for the final coil results in a spring with a free length deflection of 49.7 mm and which only occupies a volume of 17.9mm^3^. However equation (3) calculates the deflection (*f*) based on a solid length of $n\times d$ whereas the compact design of the conical spring requires the spring wires to be coiled inside each other, this will result in a greater deflection length and therefore an increase in the initial starting load. As the deflection is known the new starting load ${F}_c$ can be calculated as follows:

4 $$ {F}_c=\frac{F_d}{f}\left((nd)-d+f\right) $$

The increased spring deflection results in a new starting force $\left({F}_c\right)$ of 0.59 N however at the end of the 12.0 mm stroke length the conical spring will be delivering 0.43 N. The deflection force can be used to determine the stress (τ) in the spring and can be calculated by rearranging the following formula:

5 $$ {F}_c=\frac{\pi {d}^3}{16{r}_1}\tau $$

The 0.59 N force which is generated by the spring at the start of the stroke results in 915.2MNm^−2^ of stress induced in the compressed spring. The requirement for the spring is for a single operation therefore the maximum allowable static stresses would be limited to 59 % of the material’s ultimate tensile strength (2,200MNm^−2^) [31]. The resulting stress in the spring represents 41.6 % of the allowable static stress therefore the variable pitch conical compression spring design will be acceptable for the application.

### Trigger mechanism

**Fig. 5:**
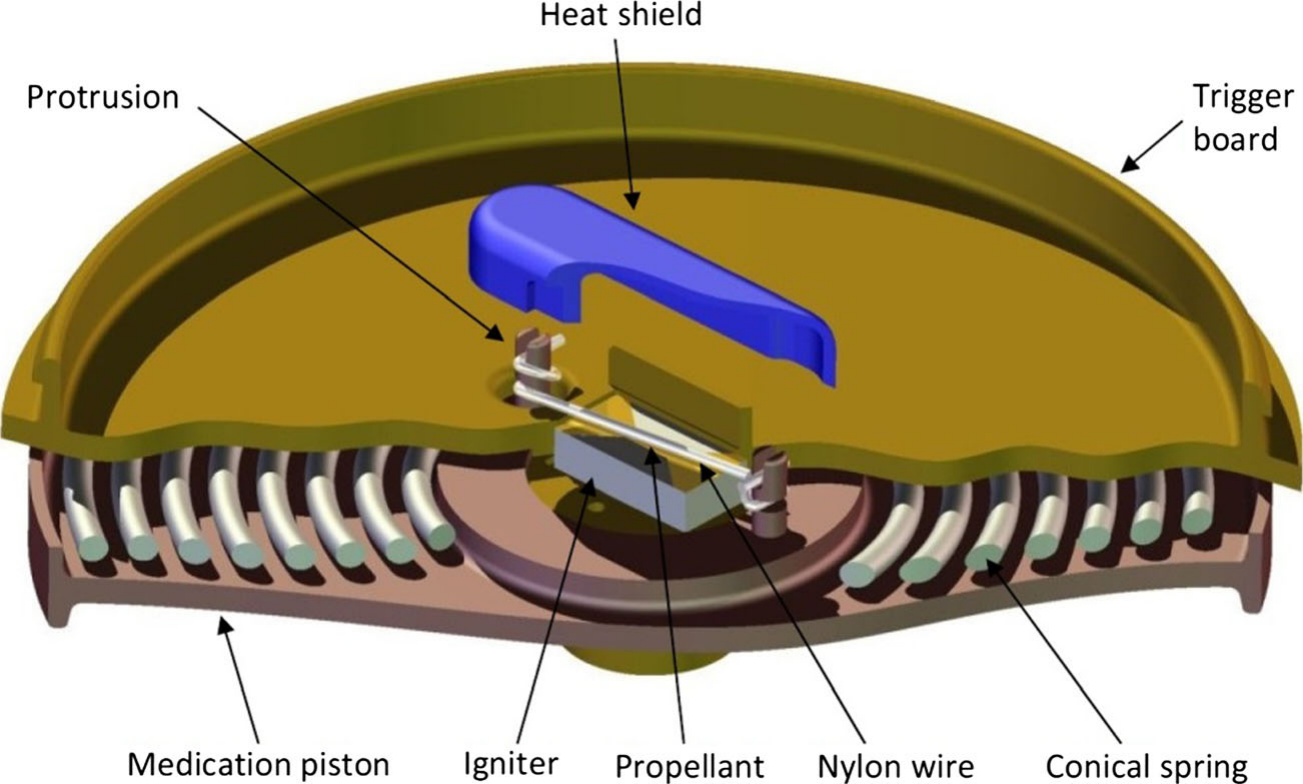
Cut-through view of the thermo mechanical trigger mechanism. Heat shield shown suspended above the igniter and Nylon wire

To deliver the 1 ml volume of medication a trigger mechanism is required to release the compressed conical spring. Releasing the spring remotely at the required moment in time is a challenging requirement. The system chosen to release the spring is a thermo mechanical method. This method of triggering the spring relies on the Joule’s effect to heat an igniter which in turn ignites a pyrogen that melts a Nylon wire. The Nylon wire is employed to retain the compressed coiled spring. Figure 5 shows a layout of the proposed system.

Figure 5 shows a section view through the thermo mechanical trigger mechanism. The trigger mechanism utilises a Nylon wire to retain the medication chamber piston (Fig. 1) which in turn traps the conical spring, preventing it from expanding. The medication chamber piston has two protrusions which pass through the trigger board. The trigger board connects the power supply section to the main body of the microrobot. The protrusions have been designed with two perpendicular tapered slots. The tapers in the slots retain the Nylon wire when it is manually wrapped around the protrusions. The Nylon wire passes over the igniter and propellant. A heat shield is placed over the top of the Nylon wire and it also encompasses the protrusions. The heat shield is retained by two vertical arms mounted on the trigger board to prevent it from separating at the point of ignition. Once the thermo mechanical mechanism has been fired the medication piston will be propelled towards the front of the microrobot, this action will simultaneously suck the exhaust fumes and heat from the propellant into the medication chamber, therefore dissipating any heat generated and preventing heat radiating into the power supply area.

The compressed conical spring will be delivering a load of 0.59 N at maximum compression therefore the Nylon wire must be capable of holding this force without breaking prematurely or elongating under the strain and partially delivering the medication. A Nylon monofilament wire has been selected which melts at 220 °C, has a Young’s modulus of 3,300MNm^−2^ and a diameter of 0.10 mm. The technology chosen to ignite the propellant, which in turn will melt the Nylon wire, is an electro-pyrotechnic initiator thin film chip (EPIC) (Vishay Intertechnology, Inc.). The microjoule energy device is a surface mount device (SMD) (size 0603) comprising a ceramic (alumina) based substrate with a tantalum nitrogen (Ta2N) deposit as the activating resistor. A propellant, which ignites at 456.8 °C [32], is applied to the EPIC igniter.

The resistance $\left({R}_{tn}\right)$ of the tantalum nitrogen heating element is dependent on the resistivity of the material and the geometry of the element. It can be calculated as follows:

6 $$ {R}_{tn}=\frac{r_{tn}l}{wt} $$

Where ${r}_{tn}$ is the resistivity of the tantalum nitrogen material (400μΩ x cm), *l* is the length of the heating element, *w* is the width and *t* is the material’s thickness.

Using the geometry of 75 μm for the length (*l*), 50 μm for the width (*w*) and 2 μm for the thickness (*t*) results in a heating element resistance $\left({R}_{tn}\right)$ of 3.0Ω. The electrical energy (*E*_*i*_) required to ignite the propellant and melt the Nylon wire can be calculated by the following formula:

7 $$ {E}_i= lwt\rho {C}_p\varDelta T $$

Where *l* is the length of the heating element, *w* is the width, *t* is the material’s thickness, *ρ* is the density of tantalum nitrogen (11.5 gcm^3^), ${C}_p$ is the specific heat of the material (0.14 J g^−1^ °C^−1^) and $\varDelta T$ is the adiabatic temperature required at the heating element.

Using equation (7) with the heating element geometry already specified and an ignition temperature $\left(\varDelta T\right)$ of 456.8 °C results in an electrical energy of 5.5 μJ required to ignite the propellant. However a safety factor is required to ensure that the electrical pulse delivered to the EPIC device is sufficient to ignite the propellant therefore a safety factor of 10 will be applied. Incorporating the safety factor into equation (7) results in 55 μJ of electrical energy required to ignite the boron potassium nitrate propellant.

The firing time $\left({F}_t\right)$ required to ignite the propellant can be calculated using the following formula:

8 $$ {F}_t=\frac{E_i}{R_{tn}{I}^2} $$

Where ${E}_i$ is the electrical energy required to ignite the propellant, ${R}_{tn}$ is the resistance of the heating element and ${I}^2$ is the supply current.

Using equation (8) with a supply current of 0.5 A results in a firing time of 74 μs. Increasing or decreasing the current will affect the firing time. However the combination of the heating element geometry and the supply current must be chosen to guarantee the device does not fire through external influences, such as electro-magnetic interference from the environment, and can only be fired by the activation energy pulse.

### Electronics

The microrobot presents a number of challenges to realising a system which can perform targeted drug delivery as limitations on space restrict the use of components, however the various electronic components and wiring must be considered. The key electronic components will include the following:

CMOS Image Sensor: This design uses a commercially available subminiature image sensor manufactured by Toshiba (TCM8230MDA) with four white SMT LEDs manufactured by Agilent (HSMW-C191) integrated on a PCB.

Controller ASIC: The power management (i.e., dc/dc conversion), telemetry, timing and control are being implemented in a full-custom ASIC in a commercially available 0.18 μm CMOS technology. Much of the specific circuit implementations will be based on previously reported systems [33–35].

**Fig. 6:**
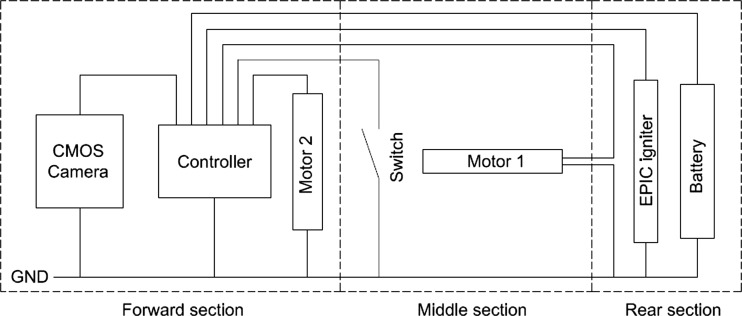
Circuit schematic of the power supply requirement for the microrobot

System Wiring: The increased functionality the mechanisms bring to the microrobot present the challenge of supplying power to the actuators performing the various operations. A circuit schematic of the power distribution for the microrobot is presented in Fig. 6.

Figure 6 highlights the requirement for multiple connections to pass through the forward, middle and rear sections of the microrobot. A more precise evaluation shows that the microrobot can be split into five distinct sections which all have a demand for power:

- The visualisation and processing section
- The holding mechanism
- The needle positioning mechanism
- The medication delivery system
- The power supply section

**Fig. 7:**
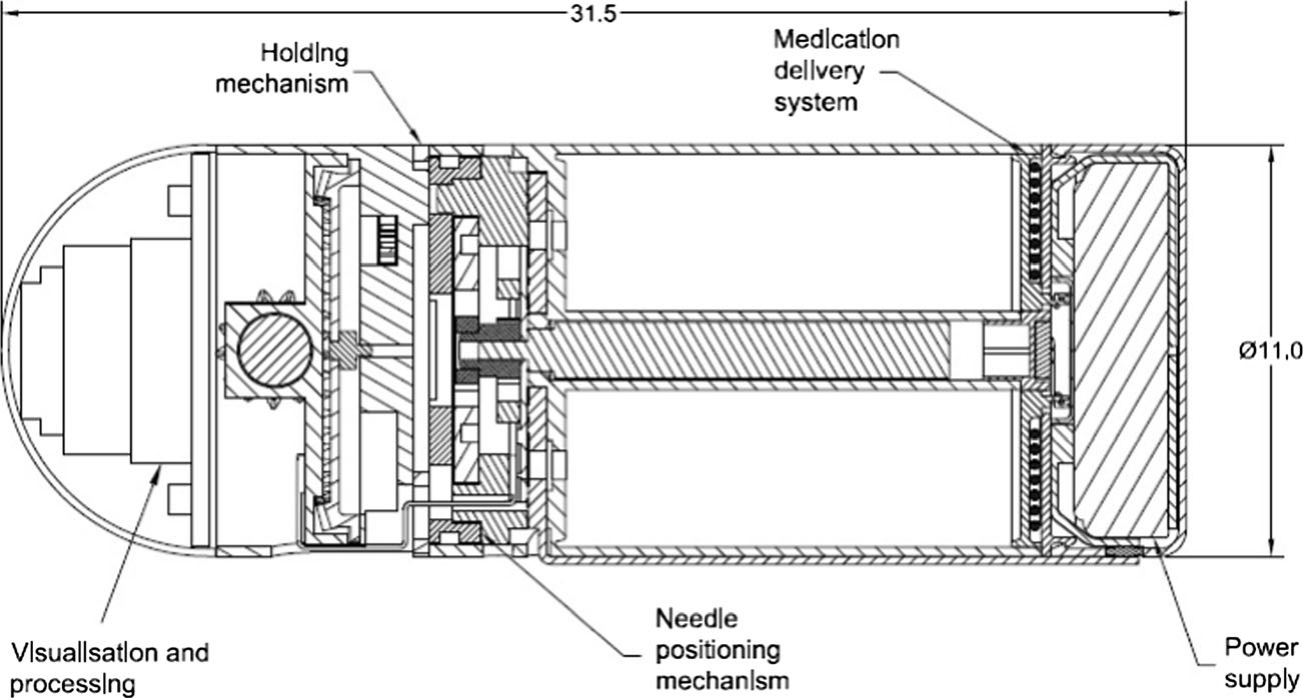
Cut-through view of the microrobot with the five main sections labelled

The configuration of the five sections of the microrobot presents problems of power delivery through the device, specifically delivering power to the holding mechanism and the visualisation and processing sections. This is due to the requirement that the holding mechanism can be deployed diametrically opposite the needle. This feature requires the front section of the microrobot to rotate 360° relative to the rear section. It is assumed that the rear section will remain static due to the mass of the medication and power supply and also from the pressure generated by the GI tract wall. The needle positioning mechanism and the medication delivery system have direct access to the power supply, however the medication chamber and the micromotor driving the needle positioning mechanism inhibit the direct passage of power to the front of the microrobot. Figure 7 highlights the five sections with a cut-through view of the microrobot.

To overcome the issue of power distribution through the microrobot and to control the angular orientation of the needle positioning mechanism a specifically designed rotary encoder is proposed.

#### Rotary encoder

**Fig. 8:**
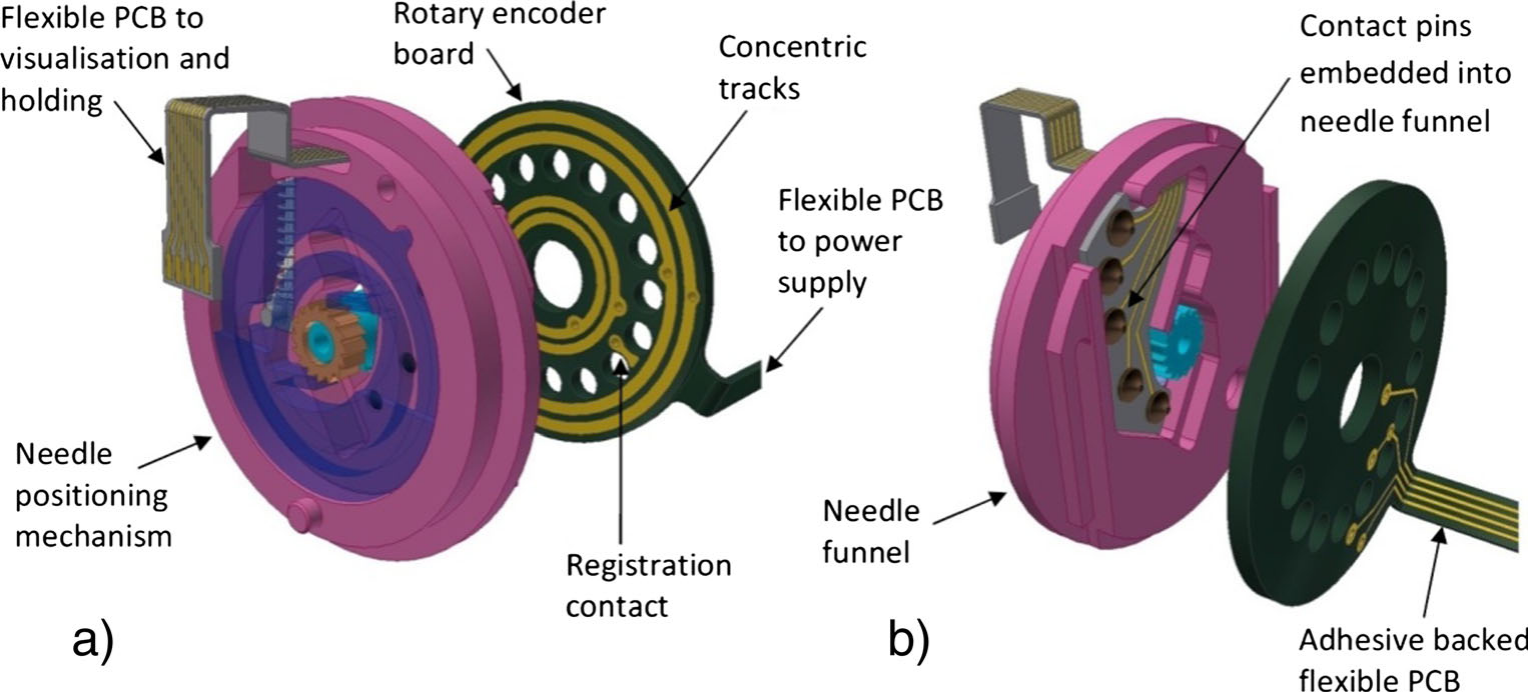
Rotary encoder: concentric tracks and registration contact (**a**) and contact pins embedded in the needle funnel (**b**)

A specifically designed rotary encoder mounted between the medication chamber and the needle positioning mechanism is the proposed solution for providing power through the forward section of the microrobot, Fig. 8. The rotary encoder uses flexible PCB cabling, which adheres to the outside of the microrobot’s body, to connect the power supply at the rear of the microrobot to the rotary encoder board. Utilising the outside of the microrobot bypasses the need to disrupt the medication compartment making sealing the medication chamber more straightforward.

The rotary encoder board, which is 0.5 mm thick, allows power to be supplied to the visualisation and processing section and to the holding mechanism while the front of the microrobot is rotating. The rotary encoder achieves this through four 0.3 mm wide concentric tracks which have been positioned to take into consideration the limitations of flexible PCB manufacture and also to accommodate the holes which allow the medication to pass through the board and into the needle funnel, (Fig. 8a). Contact pins held in the needle positioning mechanism make contact with the tracks however they remain free to rotate with the needle funnel. A fifth contact pin is mounted in the needle funnel and used in conjunction with a registration contact on the rotary encoder board to determine the angular position of the needle and holding mechanism, (Fig. 8b).

The contact pins align with pads on a flexible PCB board which is incorporated into the needle funnel. The pins stand proud of the needle funnel by 0.05 mm to ensure they make contact with the tracks on the rotary encoder board. The flexible PCB passes through the needle funnel and through the retaining plate.

The shape of the distal end of the holding mechanism’s legs have been designed to accommodate the flexible cable, however the cable cannot pass through the bevel gear set which is positioned behind the holding mechanism’s legs as it will be rotating. Therefore the power supply is again routed out through the side of the microrobot and then back into the space behind the bevel gear set where it can supply power to the visualisation and processing section and to the holding mechanism.

The power supply for the forward section is supplied through the onboard battery which is stored at the rear section of the microrobot. As specified the rotary encoder’s flexible PCB board adheres to the outside of the microrobot’s body and connects to the power supply, however the power supply is required to be removable to allow access to the medication chamber for loading.

#### Power supply

**Fig. 9:**
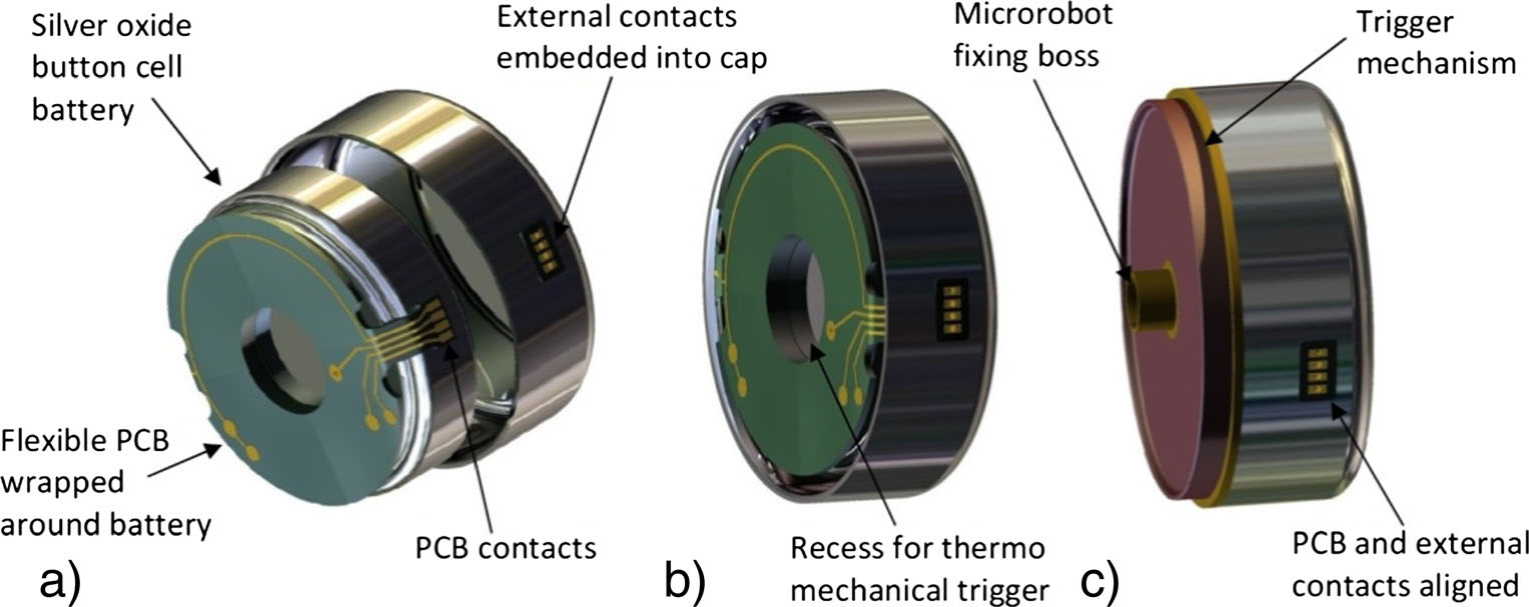
Power supply assembly: flexible PCB board wrapped around the battery (**a**) battery and flexible PCB board secured into the cap with contacts aligned (**b**) and trigger mechanism and power supply assembly ready to be loaded into the microrobot’s body (**c**)

A compact method of connecting the power supply to the microrobot is required which will allow access to the medication chamber for filling and also connect with the flexible PCB board which links to the rotary encoder. Figure 9 shows the chosen design in three stages of assembly.

Figure 9 shows the proposed assembly of the power supply unit. The solution is a modular system which allows the medication chamber to be manually loaded and also provides a method of connecting to the rotary encoder PCB board running on the outside of the body. The solution incorporates a flexible PCB board which wraps around a silver oxide button cell battery (CR1025 Energizer), (Fig. 9a). The battery and flexible PCB board are secured into a cap with resin to ensure the unit cannot be damaged. A contact board is also secured into the side of the cap which facilitates the connection to the rotary encoder PCB board, (Fig. 9b). The cap and battery are fitted to the trigger mechanism via a snap fit on the trigger board, (Fig. 9c). The assembly can be loaded onto the microrobot’s body via a central lug protruding from the trigger board. The lug secures the power supply unit and also connects the power supply to the micromotor and to the EPIC device. The flexible PCB board running on the outside of the body is connected to the contact board to complete the connection with the front of microrobot.

The proposed solution allows the medication chamber to be manually filled at the point just prior to administration. This feature gives flexibility in the choice of medication which can be prescribed to the patient therefore potentially broadening the scope of its use.

#### Energy requirements

The design of the modular battery unit allows power to be supplied to the mechanisms of the microrobot. As the onboard space to hold the power supply is limited a review of the power consumption of the various mechanical mechanisms has been performed. The energy required to operate the trigger mechanism is very low at only 55 μJ however the energy required to operate the needle positioning mechanism and the holding mechanism is much greater. The energy required to operate the two mechanism has been estimated from the peak current consumption of the Namiki motor controller (SSD04) and the Faulhaber motor controller (BLD05002S), which are stated as 800 mA at 6 V and 400 mA at 7.5 V. Three revolutions of the Namiki micromotor are required for positioning and deployment of the needle; this is achieved in 3.8 s, while the holding mechanism can complete a full opening and closing cycle in 1.78 s. Using a Lithium coin battery with a volume of 188mm^3^ and an energy density of 435 Wh/L (CR1025 Energizer), the mechanisms will consume at most 28.94 J of energy for each complete cycle which is only 9.83 % of the available energy, offering the potential for multiple operations.

## Measurement and characterisation of the medication delivery mechanism

This section presents a series of experiments designed to validate the medication delivery system. The experiments focus on determining the parameters required for the delivery of the medication and on the performance of the conical compression spring through quantitative assessment of a 1:1 scale model prototype manufactured from stainless steel (BS 2056 Austenitic 302).

### Medication delivery performance protocol

The purpose of the medication performance experiment is to determine if the chosen delivery speed of 3.5 s is suitable to expel the 1 ml of onboard medication. The analysis performed in Section 3.1 (Eq. 1) was based on water at body temperature (36 °C) however ex vivo experiments will be performed at room temperature therefore the viscosity of the water will increase to 1 × 10^−3^ Pa s to compensate. The implication of this change will be a reduction in delivery speed to 5 s to maintain the calculated delivery force.

**Fig. 10:**
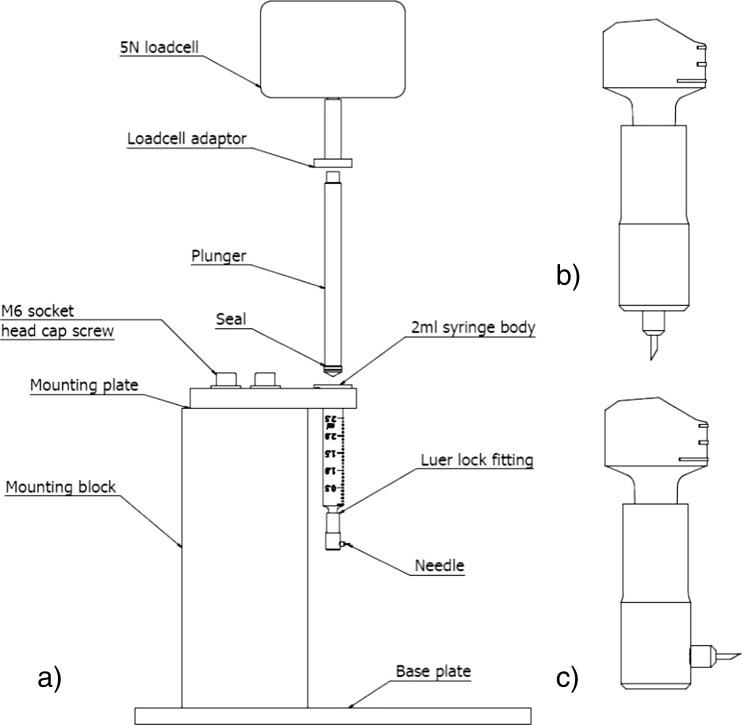
Layout of the medication performance delivery test fixture (**a)** vertical needle configuration (**b**) and horizontal needle configuration (**c**)

The test set-up used to measure the delivery force required to expel the 1 ml of medication is presented in (Fig. 10a). A specially designed plunger has been produced which can be used with a conventional syringe body. The plunger is independent of the loadcell adaptor and any sideways movement between the plunger and body has been minimised for the purpose of reducing friction within the system. The reduction in friction will result in a consistent force being generated by the plunger assembly.

The mathematical model (Eq. 1) presented for calculating the force required to deliver the medication assumed a straight through needle orientation and presented no difference between a vertical and horizontal configuration due to the Reynolds number being very low, however comparing the flow of a liquid in the vertical configuration (Fig. 10b) with the flow of liquid in the horizontal configuration (Fig. 10c) will prove the validity of this hypothesis.

#### Medication delivery procedure

Figure 10a shows the test set-up used to determine the force required to deliver a 1 ml volume of water. The body of a 2.5 ml capacity syringe (300,185, BD Plastipak) is securely held in a fixture. The bore of the syringe (Ø8.60 mm) is used to calculate the linear movement of the plunger (17.2 mm) required to deliver 1 ml volume of liquid. A 5 N loadcell (Intelligent loadcell ILC 879–010, ±0.1 % of full scale) is fitted to the linear actuator of a MultiTest 2.5-*i* (805–102) test system manufactured by Mecmesin to measure the load required for a delivery. The loadcell has been programmed to travel at the required delivery speed of 207 mm/min for a distance of 17.20 mm from the tared zero position. A computer running Emperor™ (force) software (v. 1.18–408 (5/10/13)) is connected to the MultiTest system and used to record the delivery data.

The delivery program will be run multiple times with no liquid being delivered and the data for each run recorded. The results of the average delivery force will be combined and the average of these runs will be used to calculate the friction in the system due to the seal on the plunger. The syringe will be filled with water and a piece of chicken breast flesh positioned such as the needle tip is embedded into the chicken by approximately 1.5 mm. Chicken breast flesh has been chosen as it has significant similarities to the tissue of the GI tract wall [36]. The delivery program will be run multiple times with the data for each run recorded. The results of the average delivery force will be combined and the average of these runs will be used to calculate the delivery force required to deliver the 1 ml volume of water.

#### Results and discussion

Measurement of the force required to operate the plunger assembly with the luer lock and needle fitted were first taken to establish a baseline friction force for the plunger assembly. Due to the sensitivity of the measuring system and the variability of the results a large number of deliveries were performed. Over a set of 30 runs the average delivery force for a dry delivery was 2.3 N.

**Fig. 11:**
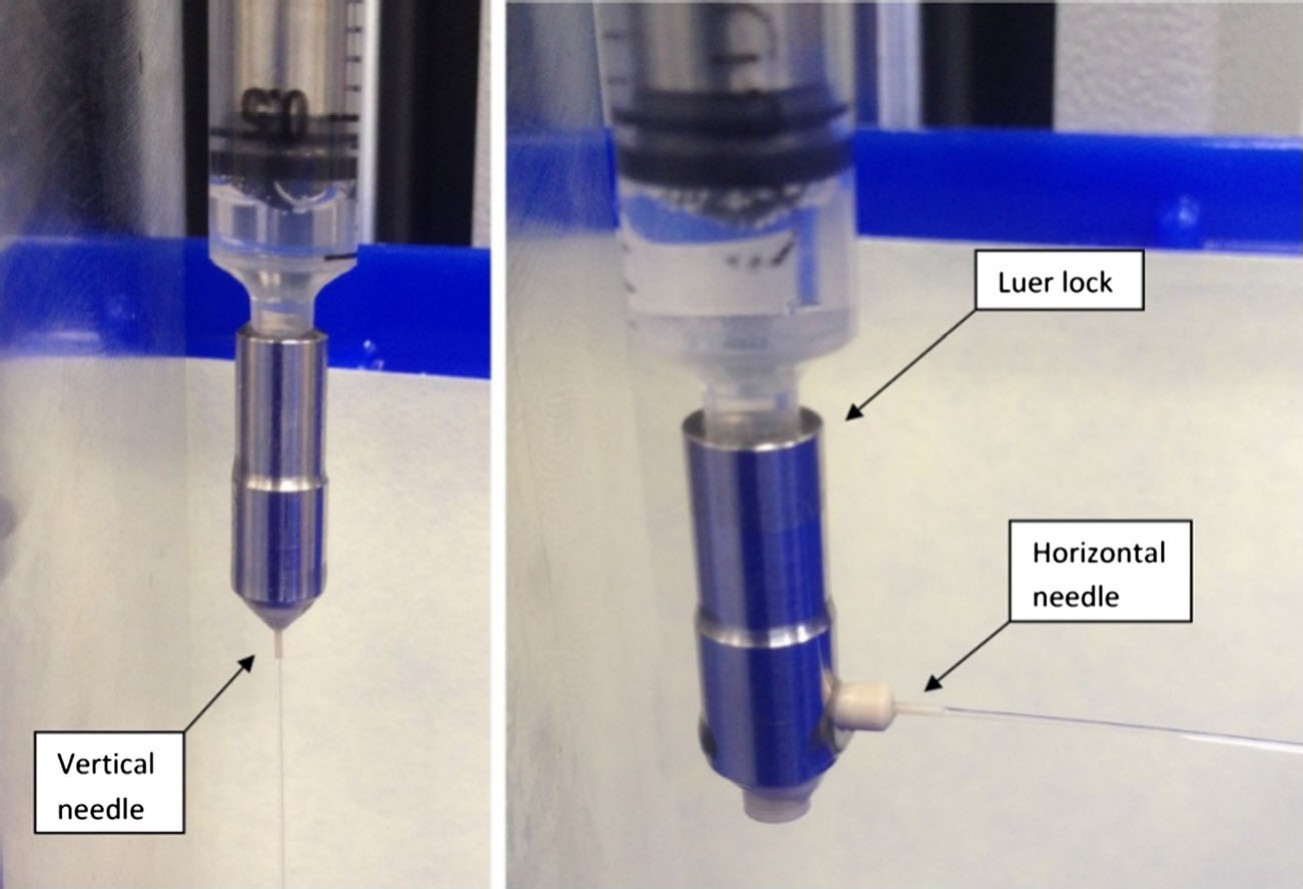
Left: Vertical needle configuration; Right: Horizontal needle configuration

A series of deliveries were performed which expelled a 1 ml volume of water with the needle orientated in the vertical configuration and also with the needle orientated in the horizontal configuration, as this would more closely match the needle orientation in the microrobot. The needle configurations can be seen in Fig. 11.

The average delivery force for a 1 ml volume of water over 10 runs for the vertical configuration was 2.75 N, this can be compared to the average delivery force for the same volume of water over 10 runs for the horizontal configuration which was 2.73 N. The results show that the average horizontal delivery force is lower than the vertical delivery force. The small difference in delivery force (17.0mN) can be attributed to the sensitivity of the test method. However the results confirm that there is very little turbulence in the system and hence confirms the low Reynolds number and that the flow will be laminar.

**Fig. 12:**
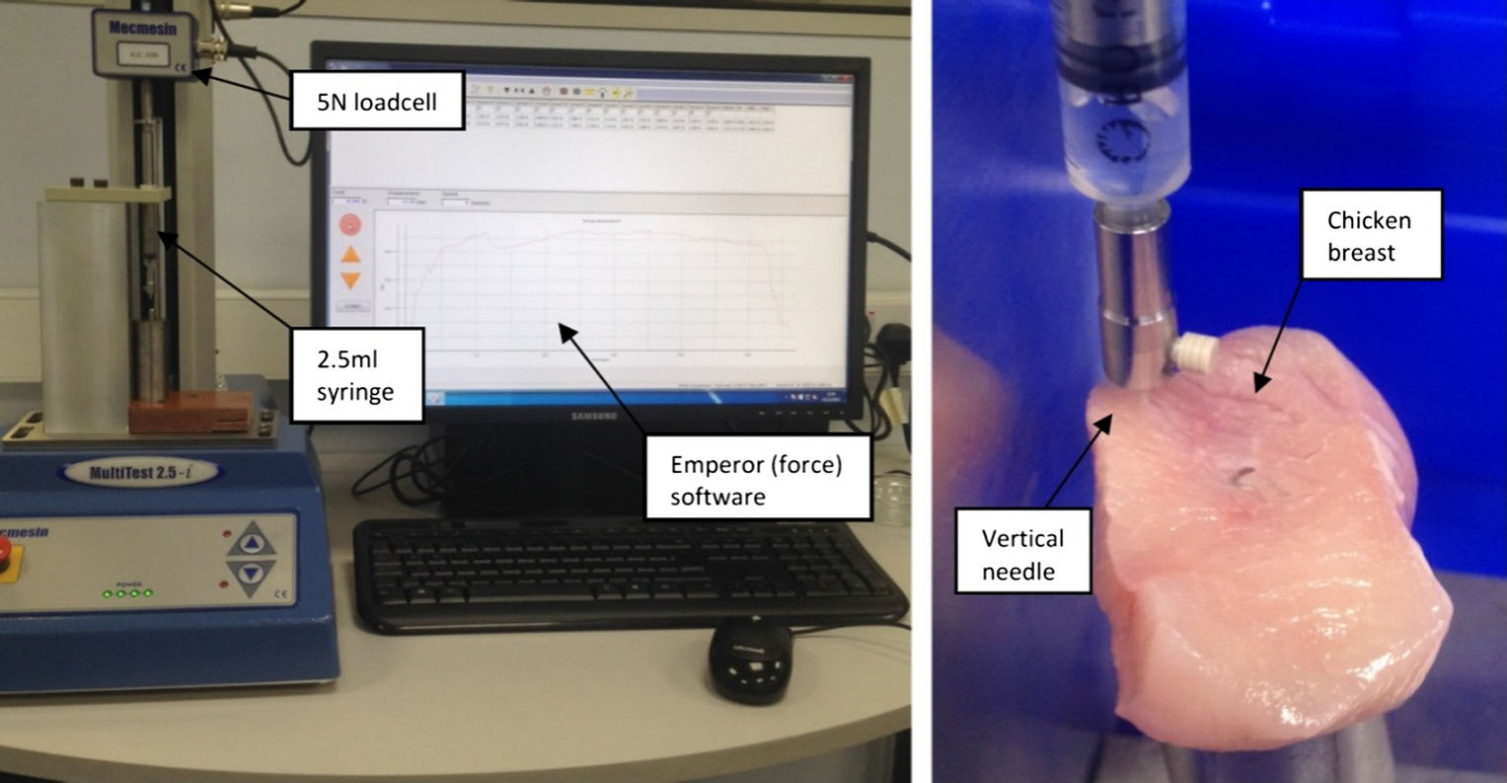
Left: Medication delivery force experimental set-up comprising: a 2.5 ml syringe body mounted on a block, the Ø0.38 mm bore needle fitted to the syringe and a MultiTest 2.5-*i* test system fitted with a 5 N loadcell and connected to a computer running Emperor™ (force) software; Right: Needle delivering liquid into a chicken breast test piece

A series of deliveries were performed which expelled a 1 ml volume of water into chicken breast flesh to simulate the intestinal pressure at the end of the needle tip. The needle was orientated in the vertical configuration as it guaranteed needle penetration and prevented the chicken breast flesh from being pushed away from the needle due to the pressure of the delivery force. The set-up used to measure the delivery force can be seen in Fig. 12.

**Fig. 13:**
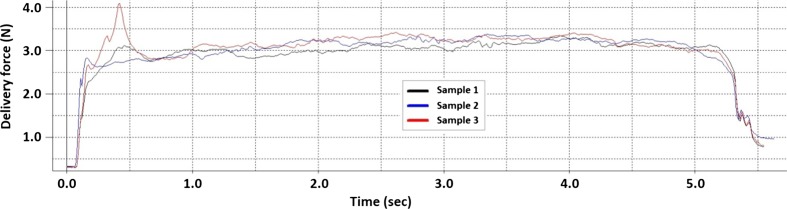
Overlay of three plots of the measured delivery force required to deliver a 1 ml volume of water into chicken breast flesh

An overlay of the measured load profiles for three deliveries of a 1 ml volume of water into chicken breast flesh can be seen in Fig. 13. The average delivery force required to expel the volume of water was 2.95 N with a standard deviation from the mean of 40mN. However subtracting the baseline friction force for a dry delivery of 2.32 N from the delivery force results in a delivery force of 0.63 N. This figure is very close to the calculated figure of 0.59 N.

The test result plots highlight the behaviour of the delivery method, for example a spike of 4.0 N can be seen in sample 3 at the beginning of the delivery (Fig. 13). The spike can be attributed to the build-up of liquid due to the chicken breast flesh not absorbing the water quickly enough and hence causing a back pressure. The measured data suggests that the delivery speed of 5 s should be increased to allow the medication to penetrate and be absorbed by the tissue however this is based on the behaviour of chicken breast flesh at room temperature and in vivo tests would need to be performed to validate the actual figures.

### Spring deflection protocol

To deliver the medication in 5 s the variable pitch conical compression spring must be capable of providing the calculated force of 0.59 N at the start of the delivery. Therefore the design of the spring must be validated to ensure its suitability to perform the task. There are no British Standards or industry standards for the design of conical compression springs however The Institute of Spring Technology (IST) provides a software package which is based on theoretical mathematical models and empirical data to aid in the design and analysis of conical compression springs and also to determine their suitability for manufacture. The IST software will be used to verify the calculations derived in Section 3.2.

The space available onboard the microrobot to house the spring has changed due to the requirements imposed by the trigger mechanism therefore the spring parameters will be modified from those used on the theoretical mathematical model (Eq. 4) to accommodate the available space. Using the IST spring calculator professional software (v1.0.18) a prototype 1:1 scale variable pitch conical compression spring will be designed and evaluated for its suitability to deliver the required starting load and also to perform within acceptable stress levels (59 % of UTS).

A stainless steel wire spring (BS 2056 Austenitic 302S26 G2) with a module of rigidity value of 70.3GNm^−2^ has been selected for the prototype spring. The 0.25 mm diameter wire has 7.5 active coils, 2 dead coils for the purpose of stabilising the spring during operation, a mean coil radius of 2.6 mm for the first coil and a mean coil radius of 5.12 mm for the final coil and a free length of 48.8 mm. The modified spring parameters result in a linear spring rate of 10.9mN/mm which equates to a theoretical solid state load of 0.53 N however the software predicts a solid state load of 0.79 N and a solid state stress of 991.0Nmm^−2^ (45 % of UTS). The discrepancy in load can be attributed to one of the coils prematurely touching at the final stage of compression, this will result in the coil collapsing which in turn will increase the spring rate. However it is anticipated that manual modification of the spring end will eliminate this issue and result in the calculated load (0.53 N).

#### Spring deflection procedure

**Fig. 14:**
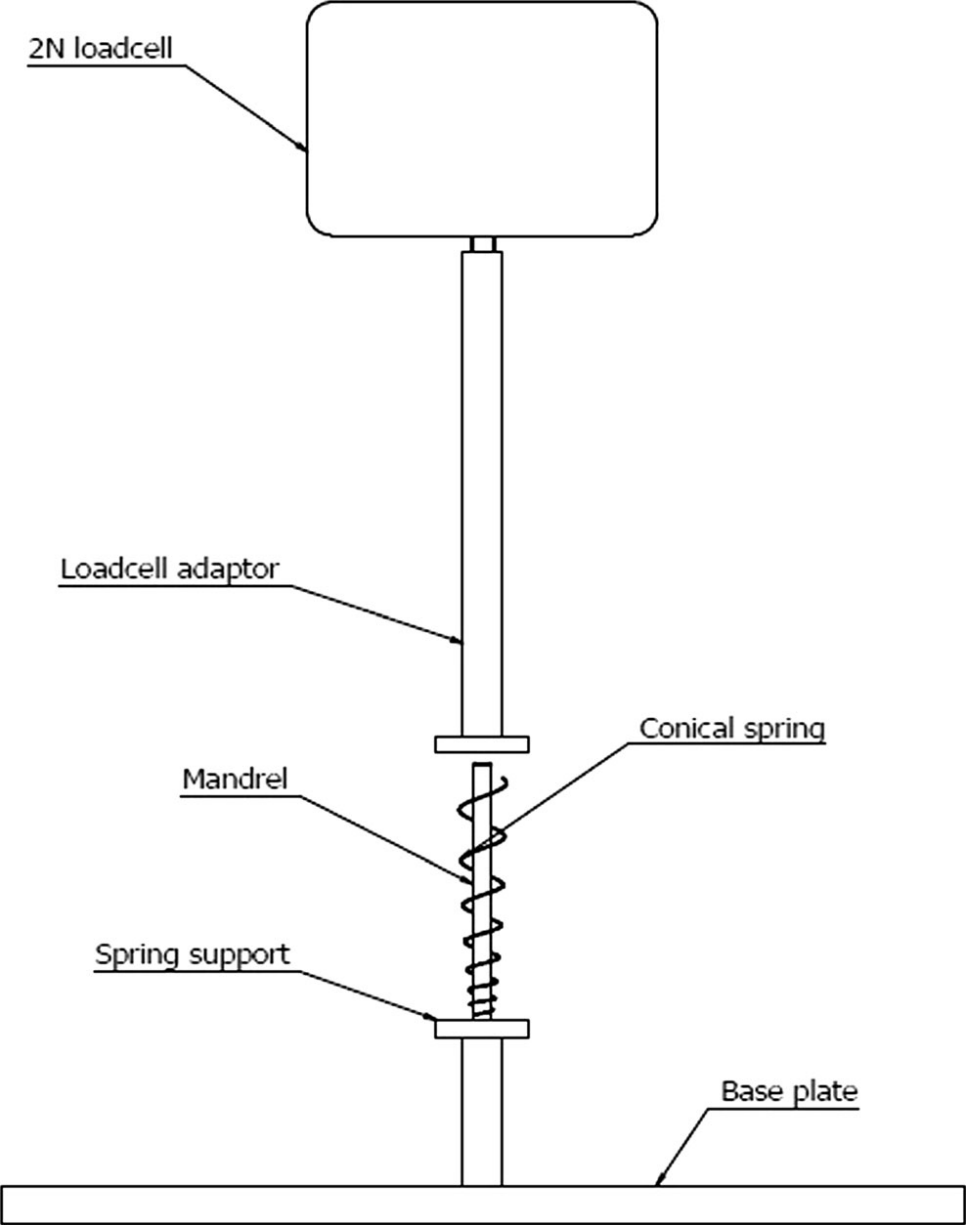
Variable pitch conical compression spring deflection load test fixture

The stainless steel conical compression spring will be mounted on a mandrel and held between two supports which have been designed to hold the end coils in position while the spring collapses. The test set-up used to measure the force delivered by the compression of the conical spring is presented in Fig. 14.

The spring will be tested on a MultiTest 2.5-*i* manufactured by Mecmesin. A 2 N loadcell (Intelligent loadcell ILC 879–009, ±0.1 % of full scale) will measure the load generated by the compression of the spring from its free state to its solid state. The test will be run at a velocity of 145.0 mm/min which matches the calculated delivery speed. The compression program will be run multiple times with the data for each run recorded. The results of the average peak force at solid state will be combined and the average of these runs will be used to calculate the delivery force. A further measurement at 12.0 mm deflection will be recorded and the average force from the deliveries will be used to determine the spring’s force at the end of its stroke.

#### Results and discussion

**Fig. 15:**
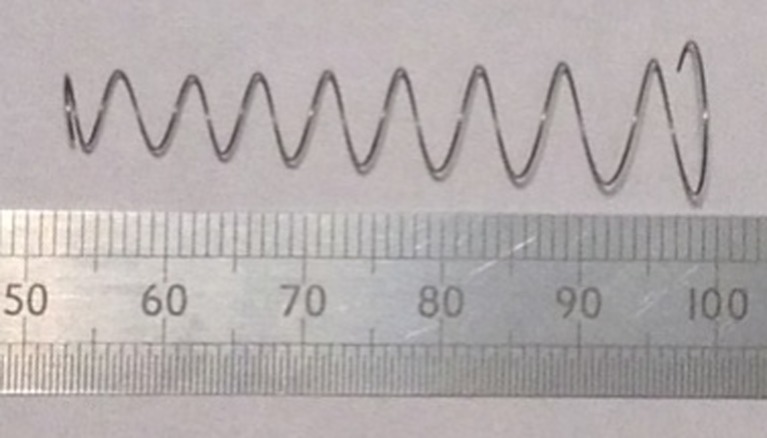
1:1 scale prototype stainless steel variable pitch conical compression spring

A total of 10 springs were dimensionally inspected on a digital vision measuring system (TESA Vision 200, TESA Technology UK Ltd.) with the results showing that the 2.6 mm mean coil radius for the first coil was outside of the design specification at 2.76 mm and the springs’ free length was 47.7 mm compared to the design value of 48.84 mm. The reduced clearance between the coils due to the larger radius of the first coil resulted in the coils touching during the final stages of compression. The coils could not be manually modified to reinstate the clearance as initially anticipated therefore the springs were tested through a stroke length of 46.65 mm to minimise the impact of the coils colliding. Figure 15 shows the conical compression spring in its free state.

**Fig. 16:**
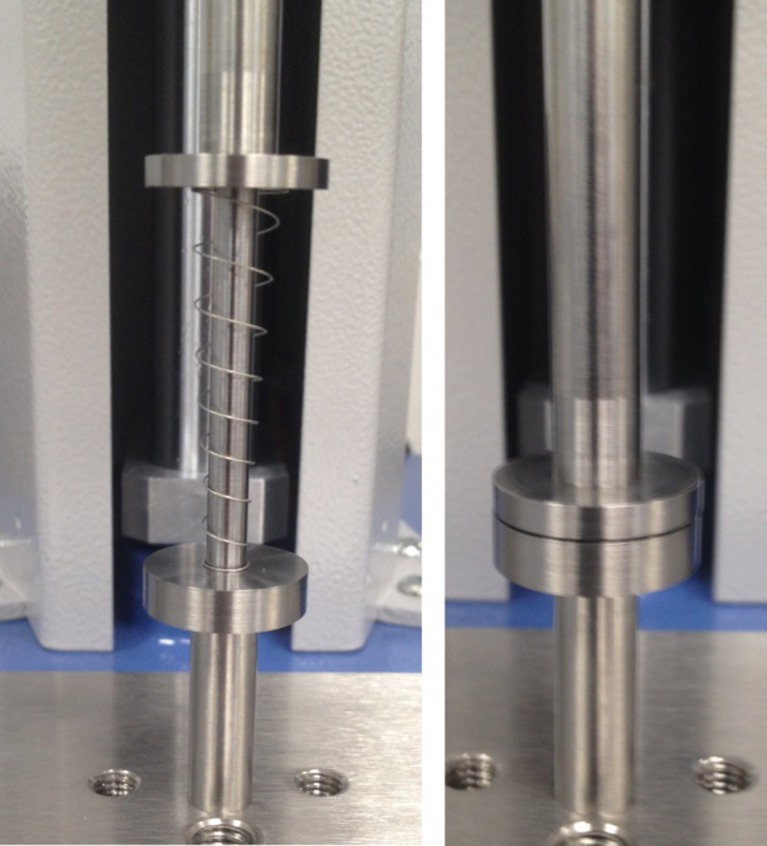
Left: Conical spring held vertically between two supports and further supported via a mandrel; Right: Spring in the fully compressed configuration

The test jig was configured such that the mandrel could pass freely in and out of the loadcell adaptor without generating a false reading on the loadcell due to friction. A 0.5 mm by 10.3 mm diameter counter bore was positioned in the loadcell adaptor support to accommodate the large coil and to prevent it from moving during compression. The 0.5 mm depth of the bore allowed clearance for the coils at the end of the stroke. Figure 16 shows the test set-up used to hold the spring vertically between two supports and via a central mandrel. The spring is also shown in the fully compressed configuration.

**Fig. 17:**
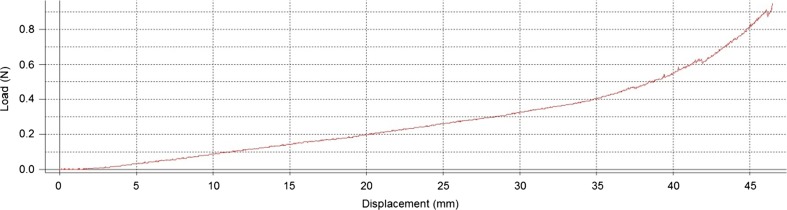
Load profile of a typical spring compression

A total of 10 springs were compressed 10 times each from a preloaded compression of 0.2 mm through a stroke length of 46.45 mm to give a total working compression of 46.65 mm. The stroke length allowed 1.05 mm of clearance at the end of the stroke for the solid state of the spring. The springs were preloaded to stabilise the coils during the compression sequence and to accommodate slight variations in the overall length of the spring. A typical load profile of a spring compression can be seen in Fig. 17.

Figure 17 shows a peak load of 0.94 N at the end of the stroke. As the coils could not be modified manually to eliminate touching during the compression the measured result can be compared to the calculated figure of 0.79 N. The higher result can be directly attributed to the coils contacting each other and increasing the spring rate. Figure 17 highlights the change in spring rate through the compression sequence. Between zero and 30.0 mm the spring rate is 10.93mN/mm resulting in a peak load of 0.32 N and between 30.0 mm and 46.45 mm the spring rate significantly increases to 37.60mN/mm, resulting in the peak load of 0.94 N. The results for the series of tests show that the springs are consistent with an average spring rate of 10.97mN/mm for compression up to 30.0 mm and 32.8mN/mm up to 46.45 mm. The abrupt changes in load which are evident at 41.85 mm and 46.1 mm are due to the coils slipping over each other.

It is a requirement that the spring can deliver a force of 0.43 N at the end of its 12.0 mm stroke to guarantee delivery of the medication in 5 s. It can be seen in Fig. 17 that a load of 0.42 N is developed at 35.65 mm compression which represents the end position of the piston (12.0 mm). The results of the protocol show that the conical compression spring will deliver sufficient force to expel the medication in 5 s and that the stress in the spring will be at a safe level as the overall length of the springs remained the same after repeated operations. The performance of the spring could benefit from some improvements. For example, generally increasing the clearance between the coils, reducing the number of coils and increasing the wire diameter would result in a more stable spring which would deliver a consistent load and have lower levels of stress. However the deliverable force at the end of the 12.0 mm stroke would drop off slightly resulting in a longer delivery time.

## Conclusion

In this paper we have presented the first WCE with increased functionality to allow targeted drug delivery to be performed in the small intestinal tract. A novel drug delivery system has been proposed which delivers 1 ml of medication and only occupies a volume of 65.0 mm^3^.

It has been shown through analysis and experimentation that a stainless steel variable pitch conical compression spring can be wound such that it has a solid state thickness of the wire diameter and occupies a volume of 17.9mm^3^. The conical spring is capable of delivering a force of 0.59 N at the solid state and a force of 0.43 N at the end of the 12.0 mm stroke which is sufficient force to expel the 1 ml of onboard medication in the required delivery time of 5 s.

The presented solution for targeted drug delivery combines a holding mechanism capable of resisting peristalsis, a targeting mechanism capable of positioning a needle within a 360 degree envelope and a medication delivery system and integrates them into a conventional wireless capsule endoscope. The combined mechanisms only occupy 75 % of the available volume including the 1 ml of medication and the power supply.
